# Microplastics in sea ice drifted to the Shiretoko Peninsula, the southern end of the Sea of Okhotsk

**DOI:** 10.1038/s41598-024-78108-9

**Published:** 2024-11-27

**Authors:** Hiroshi Ohno, Yoshinori Iizuka

**Affiliations:** 1 Kitami Institute of Technology, Kitami, Hokkaido Japan; 2 Institute of Low Temperature Science, Hokkaido University, Sapporo, Hokkaido Japan

**Keywords:** Environmental sciences, Ocean sciences

## Abstract

Sea ice is regarded as a temporal sink and carrier of microplastics (MPs). Nevertheless, knowledge and understanding of MPs in sea ice remain sparse. This study investigated the abundance, composition, size (> 30 μm), and shape of MPs in four sea-ice cores retrieved at the southern end of the Sea of Okhotsk. Nine microplastic (MP) types, mostly with fragmentary shapes, were detected among ice-core sections. Most fragmentary MPs were smaller than 120 μm, but all fiber MPs were in the largest size class (> 210 μm). MP concentrations were 0–60 particles/L, with an average of 21 particles/L. Higher occurrences of MPs observed in the lower ice layers are attributable to heavier MP contamination in the southern part of the sea and/or relocation of MPs in the ice matrix. No significant correlation was found between the distributions of MP and inorganic particle (sediment) abundances, implying difference in their kinetics of suspension freezing. Taken together, these findings suggest important implications for elucidating the nature and distribution of MPs in sea ice.

## Introduction

Growth of global plastics production since the 1950s has greatly outpaced that of any other manufactured material^1^, reaching approximately 400 million metric tons (Mt) per year at present^2^. Reportedly, approximately 100 Mt of plastic waste was generated in 2010 by people living in 192 coastal countries (93% of the global population)^3^. It has been estimated that approximately 5–13 Mt of this waste entered the ocean in 2010, equivalent to approximately 2–5% of the total annual plastic production^3^. Although anthropogenic plastics are in most cases not biodegradable, they are subject to fragmentation by mechanical abrasion and UV degradation in natural environments^4^, consequently disintegrating into tiny particles (< 5 mm) as so-called microplastics (MPs). Uptake and accumulation of MPs and their related chemicals in organisms are regarded as hazardous for ecological systems^5^ and for human health^6^. Moreover, recent studies have underscored the possible effects of MPs on global carbon and nitrogen cycles^7,8^.

In the course of investigating Arctic sea ice as a habitat for diatomaceous life, Obbard et al.^9^ unexpectedly discovered extreme enrichment of MPs in ice. They suggested sea ice as a major historic global sink of MPs. Subsequently, several studies^10–15^ have confirmed that MP concentrations in sea ice are orders of magnitude higher than those in surface seawater everywhere in ice-covered seas. Reportedly, the estimated amount of plastic particles (sum of MPs and mesoplastics) entrapped in sea ice of the western Arctic Ocean based on seasonal sea-ice coverage data and plastic-particle concentrations obtained from ice core analyses is comparable to that of MP stocks afloat in the global oceans^14^. Because sea ice can drift far from where it formed^16^, and because the Arctic Sea ice volume has declined in recent years^17^, it is regarded as a vector and secondary source of MPs^9,18^. Field observations on MP abundances in seawater and sea ice from Svalbard have supported the idea of considerable MP release from sea ice during summer ice melting^11^. Because this MP release and the ice-edge bloom occur simultaneously and in the same place, some concern has arisen about the intake of MPs by organisms^11^. A recent report described high levels of MPs in Arctic sea ice algae^19^.

Nevertheless, because of scant field data^9–15,20,21^, little is known about the features and pathways of MPs in sea ice. To elucidate the nature and origin of MPs in sea ice, we investigated the abundance, polymer type, size, and shape of MPs in sea ice drifted to the Shiretoko Peninsula, the southern end of the Sea of Okhotsk, using techniques based on microscopy and micro-Fourier Transform Infrared Spectroscopy (micro-FTIR).

## Methods

### Sea-ice sampling

**Fig. 1:**
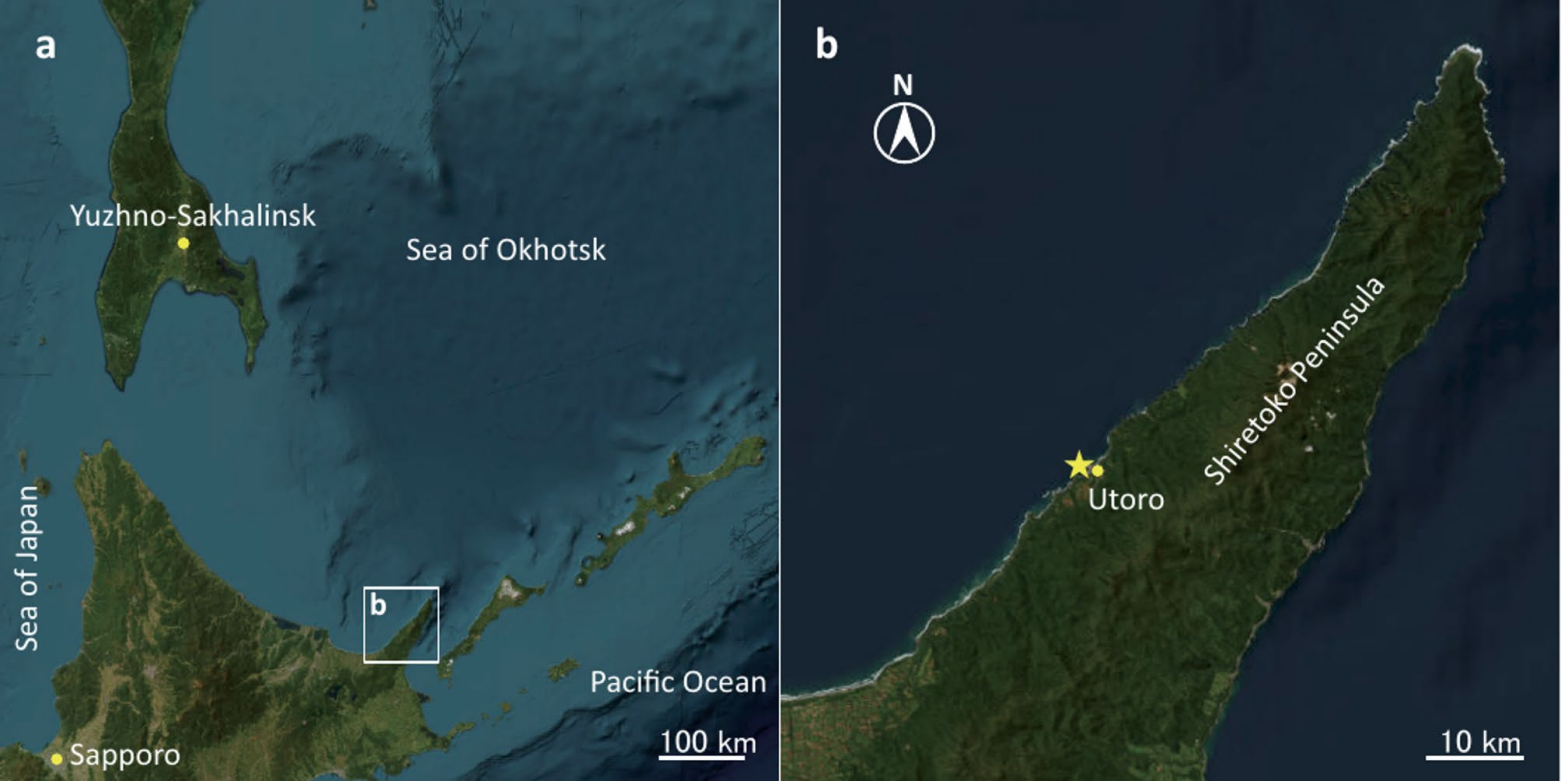
(**a**) Southern part of the Sea of Okhotsk. (**b**) The Shiretoko Peninsula. A solid star represents the location of sea-ice sampling. Maps were created using ESRI ArcGIS Pro (ver. 3.0.3, https://www.esrij.com/products/arcgis-pro/).

On 16 February 2023, sea-ice samples were collected near Utoro (Fig. 1), located on the western side of the Shiretoko Peninsula, which is known as the gateway to the Shiretoko World Natural Heritage Site. After removing snow deposited on thick (approximately 40–50 cm) fast ices (not grounded), four ice cores were retrieved by vertically penetrating the different ice blocks using a stainless-steel ice corer (8-cm inner diameter, SIPRE auger; Jon’s Machine Shop). The cores were packed immediately into polyethylene bags and were stored in a cooler box that had been precooled to − 15 °C. Within the same day, the cores were transferred to a freezer set at − 43 °C and were stored until sample preparation before measurement.

Because of the development of a surface water layer with low salinity, the Sea of Okhotsk is the southern limit of a sizable seasonal ice cover in the Northern Hemisphere^22^. Freezing in the Sea of Okhotsk normally begins in November on the northern coast of the sea in Siberia^23^. Then the ice is advected southward while growing by the prevailing northerly winds and the southward flow of the East Sakhalin Current^24,25^. The ice finally reaches the Okhotsk coast of Hokkaido, including the Shiretoko Peninsula, from late January to early February (Fig. 1). Although detailed migration routes of our ices are unknown, the ice chart provided by the Japan Coast Guard has indicated that the ice drifted to the Peninsula in early February.

### Sample preparation

Using a stainless steel saw, the outer surfaces (approximately 5 mm thickness) of ice cores were removed for decontamination^9,10,12–14^; then the remaining cores were cut into three subsections (upper, middle and lower layers; Table 1). After rinsing with approximately 400 mL of ultrapure water (18 MΩ cm)^10,13,14^, ice sections were thawed in tall glass beakers with aluminum foil lids at room temperature. Each thawed sample was filtered onto an aluminum oxide filter (47 mm diameter, 0.2 μm pore size, Anodisc; Whatman plc.)^26^ using a vacuum filtration apparatus made of glass. Salinity of the filtered water was measured using a conductivity meter (YSI Pro30; Xylem Inc.). With the filter still attached to the apparatus, approximately 100 mL of 30% H_2_O_2_ was poured into a glass funnel of the apparatus. The funnel opening was covered with aluminum foil. After the solution was left for 1 day at room temperature to remove natural organic matter adhered to MPs^27^, the solution was filtered with the apparatus. After finishing the filtration of approximately 150 mL of the ultrapure water, the filter was removed from the apparatus and was dried in a glass petri dish.

**Table 1:** Four sea-ice cores retrieved vertically from different fast ice blocks were cut into three subsections and were respectively named ‘upper’, ‘middle’, and ‘bottom’ from top to bottom. Depths of ice core subsections, filtered volumes of melted ice core subsections, salinities of melted ice core subsections, the numbers of particles (> 30 μm) on filters, abundances of MPs *N*_*mp*_ (> 30 μm), abundances of cellulosic particles (> 30 μm) *N*_*c*_, abundances of other organic particles (> 30 μm) *N*_*o*_, and abundances of inorganic particles (> 30 μm) *N*_*io*_.

Sample code	Depth (cm)	Vol. (mL)	Sal. (PSU)	*N* (particles/filter)	*N*_mp_ (particles/L)	*N*_c_ (particles/L)	*N*_o_ (particles/L)	*N*_io_ (particles/L)
Core-1_upper	0–15.5	414.5	2.5	402	10	29	116	815
Core-1_middle	15.5–31	411.8	2.1	129	9	6	75	222
Core-1_lower	31–43	333.2	4.0	66	33	24	57	84
Core-2_upper	0–13	430.7	3.7	453	32	263	53	705
Core-2_middle	13–26	437.8	3.1	56	5	21	25	78
Core-2_lower	26–36	252.2	3.6	91	20	8	44	289
Core-3_upper	0–18	554.4	3.1	167	9	42	33	217
Core-3_middle	18–33	526.5	2.4	273	36	192	41	249
Core-3_lower	33–48	478.9	3.3	102	19	11	60	124
Core-4_upper	0–15	341.8	1.3	277	24	49	146	592
Core-4_middle	15–30	404.2	2.0	189	0	23	117	327
Core-4_lower	30–50	652.6	2.4	560	60	172	206	420

### Microscopic observation

**Fig. 2:**
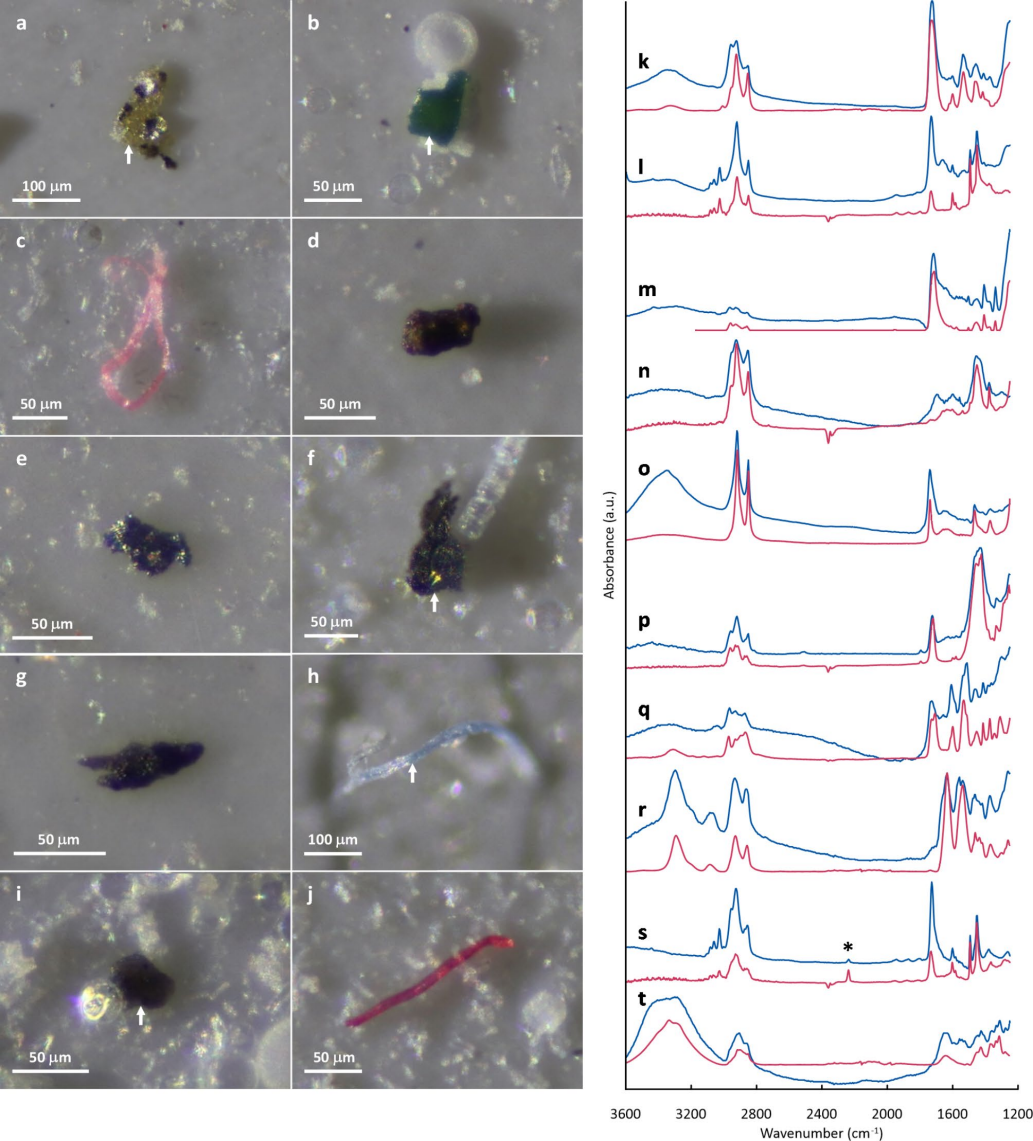
Micrographs and FTIR spectra of particles from sea-ice cores identified as (**a**, **k**) alkyd, (**b**, **l**) polystyrene, (**c**, **m**) polyethylene terephthalate, (**d**, **n**) rubber, (**e**, **o**) ethylene-vinyl acetate, (**f**, **p**) polyvinyl chloride, (**g**, **q**) polyurethane, (**h**, **r**) polyamide, (**i**, **s**) acrylonitrile butadiene styrene, and (**j**, **t**) hemp (cellulose). Spectra from the particles are shown with blue lines, whereas those for references^28–31^ are presented as red lines. Acrylonitrile butadiene styrene and polystyrene are distinguishable according to the presence or absence of the absorption peak from nitriles (asterisk in the figure).

Micropatricles on prepared filters were observed using an optical microscope (BHT; Olympus Corp.) as reported in our earlier publication^28^. To enhance the visibility of microparticles on a filter, the filter surface was illuminated laterally by white LED light from one side. For this study, only particles larger than 30 μm were examined. We counted particles except for diatom frustules, which are readily distinguishable by their transparent and geometric features, on a filter (Table 1). Additionally, after randomly selecting 100 particles for each filter as targets of micro-FTIR analysis (or all particles on a filter if the total number was less than 100), we recorded their coordinate positions on filters. The particles were classified by shape as having fragments or fibers. Targeted particles were photographed using a digital camera (EOS Kiss X3; Canon Inc.) attached to the microscope. Then their maximum diameters or lengths were measured by analyzing micrographs using image-processing software (ImageJ; NIH). Micrographs of typical particles are presented in Fig. 2a–j.

### Micro-FTIR analysis

Following our previous work^28^, chemical identification of microparticles was done using micro-FTIR. All targeted particles on filters were analyzed using an FTIR microscope (Nicolet iN10; Thermo Fisher Scientific Inc.) in transmission mode using the following parameters: 30 μm × 30 μm square field aperture, 8 cm^− 1^ spectral resolution, 64 scans, and 1250–3600 cm^− 1^ spectral range. The obtained FTIR spectra were then compared with the commercial spectral databases of standard polymers (Hummel Polymer sample Library and HR Polymer and additives) and also with open-access libraries designed for MP research, which includes spectra of aged plastics^29–32^, using spectroscopy software (OMNIC Picta; Thermo Fisher Scientific Inc.). Spectra with a match of < 60% were rejected. When returning a spectral match of > 60%, an additional visual examination of spectra was performed manually, leading to final acceptance or rejection^9,33,34^. FTIR spectra of typical particles are presented in Fig. 2k–t. Although some human-modified cellulosic fibers such as dyed hemp were detected (e.g. Fig. 2j and t), these are not classified as plastics but as celluloses for these analyses. It is noteworthy that human-modified cellulosic fibers such as rayon have been counted as MPs in some studies of marine environments^9,13,35^, but caution is necessary when identifying them because their FTIR spectra are almost identical to those of natural cellulosic fibers^36^.

### Data analysis

For this study, microparticles were classified into four compositions: MP, cellulose, other organic matter, and inorganic matter. Particles which exhibit clear IR signals within 2700–3100 cm^− 1^ (C–H stretching modes; e.g. spectra in Fig. 2), but which were identified as neither MP nor cellulose are classified as other organic matter, whereas those without C–H stretching bands are categorized as inorganic matter^28^.

**Fig. 3:**
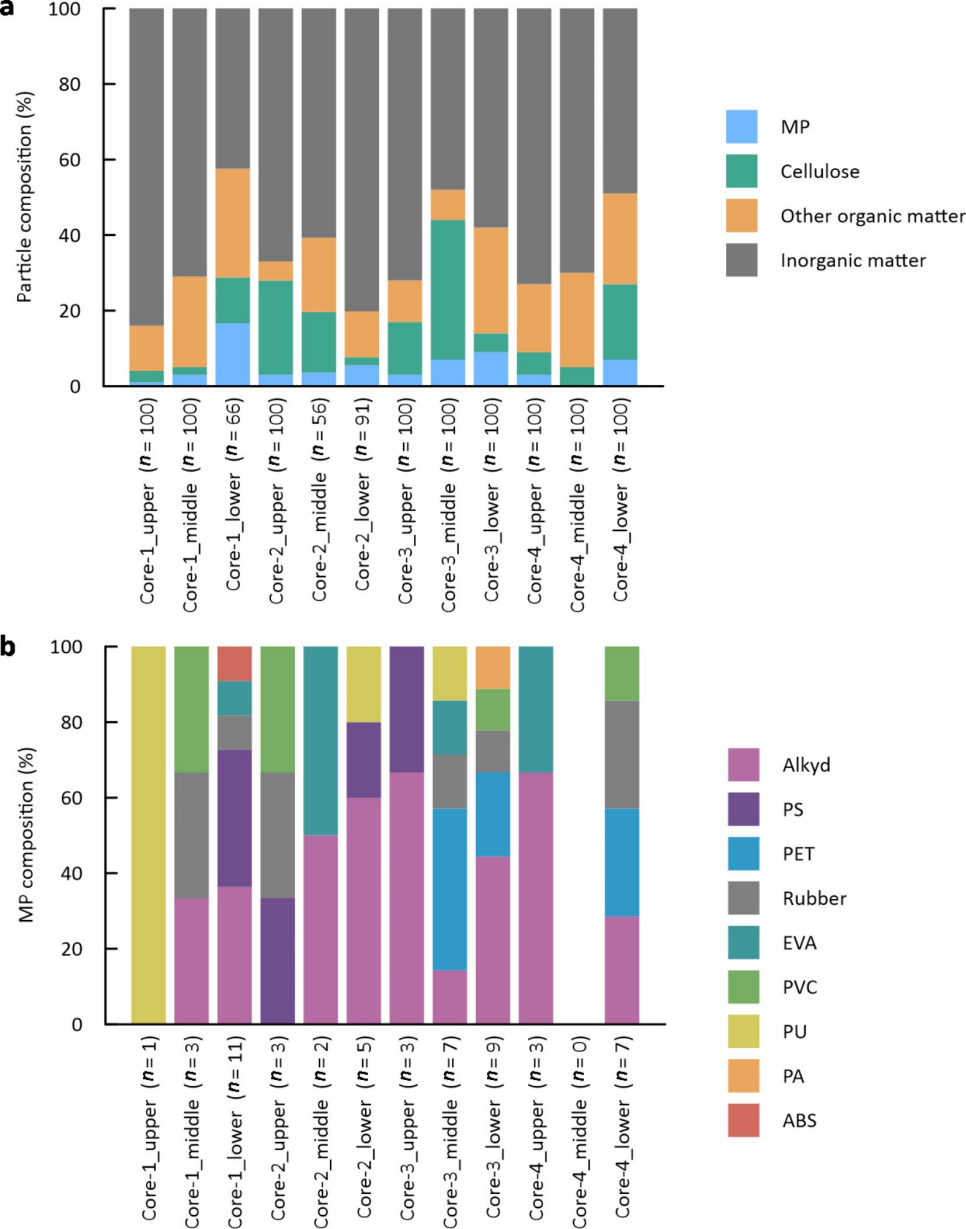
Relative compositions of particles in sea-ice cores identified using micro-FTIR. (**a**) Particle compositions and (**b**) MP compositions in ice core subsections.

Abundances of MPs in sea-ice samples per unit volume of water were estimated from the percentages of identified MPs among the total particles analyzed (Fig. 3a), the numbers of particles on filters, and the volumes of water samples (Table 1). Similarly, abundances of cellulosic particles, other organic particles and inorganic particles were also estimated (Table 1).

To elucidate the interaction of MPs with sea ice, correlations of abundances of MPs with those of other particles or sea-ice salinities were investigated using Spearman’s rank correlation analysis (Fig. 6).

**Fig. 4:**
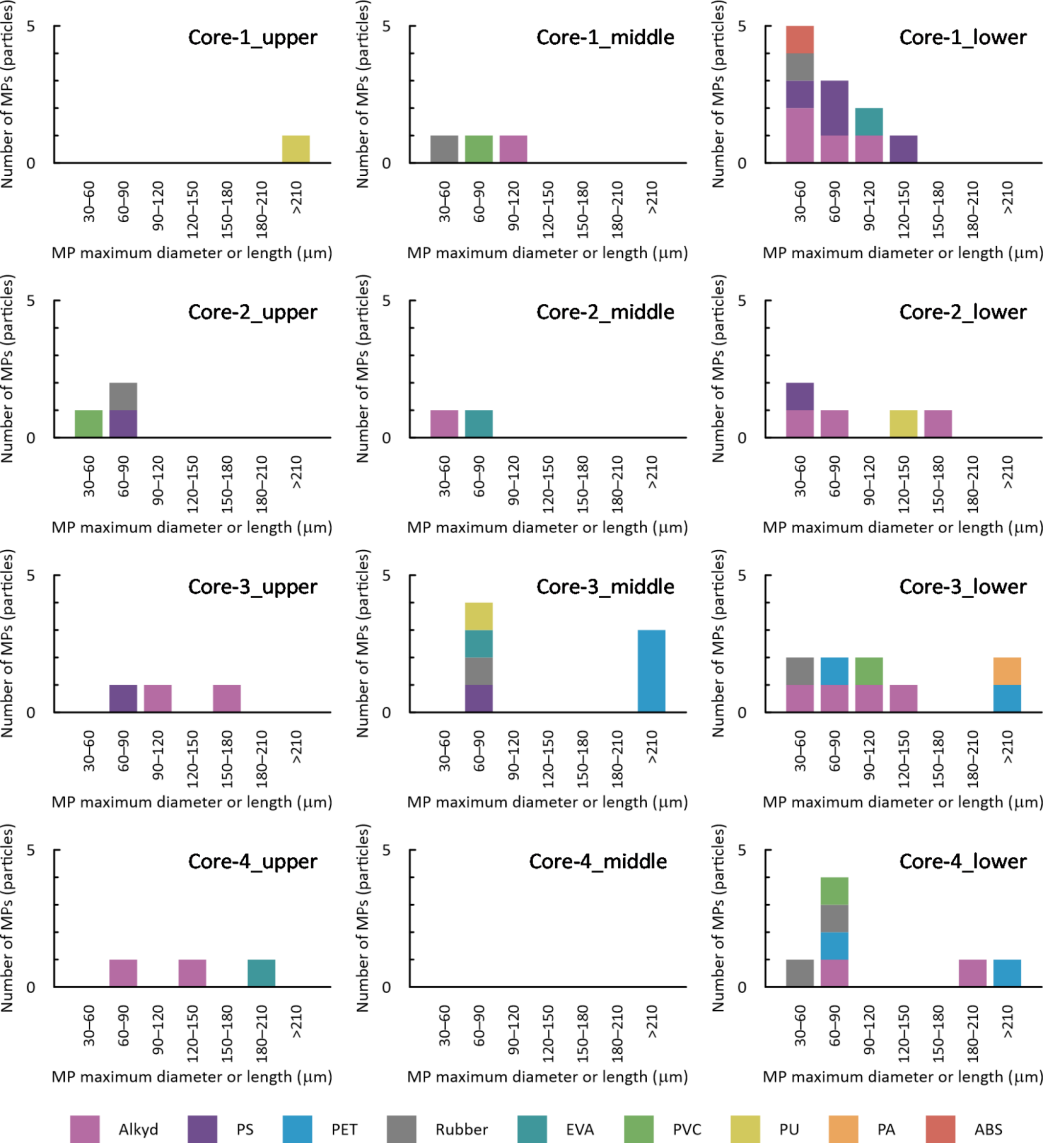
Stacked bar chart of observed numbers of MPs of various types from sea-ice cores in different size classes.

### Quality control and quality assurance

To avoid plastic contamination, all processes of sample preparation and Micro-FTIR measurements described above were performed in clean booths using HEPA filtration units. In addition, to avoid MP deposition on a sample filter during microscopic observations, a prepared filter was placed in an optical petri dish made of quartz. Then it was inspected under the optical microscope through a quartz optical window. Additionally, cotton clothes and powder-free latex gloves were always worn during all experiments.

Blank tests were conducted to assess the validity of these methods. Artificial ice cores were produced by freezing the ultrapure water in a tall stainless-steel beaker^10,12,21^. These were processed using the same procedures as those used for the sea-ice core samples. In all, three blanks were tested. Microscopic observations of blank-filter surfaces revealed several particles on blank samples. Subsequent micro-FTIR analyses of these particles detected one cellulose, one alkyd, and two epoxy (or phenoxy) resins from the first blank, one cellulose and six epoxy (or phenoxy) resins from the second one, and three cellulose and four epoxy (or phenoxy) resins from the last one. As reported from earlier work^28^, inspection of “brand-new” filters revealed that several epoxy (or phenoxy) resin particles were always present on the filter surface, irrespective of the production lot. Therefore, epoxy (or phenoxy) resin particles detected during particle analyses of the sea-ice samples were not counted as MPs from the sea ices. Except for the excluded resin particles, the averaged MP abundance on a blank filter (0.3 particles/filter) was two orders of magnitude lower than that on a sea-ice filtered filter (10 particles/filter). Therefore, almost all MPs detected in measurements of sea ices are regarded as originating from the sea-ice samples.

## Results

### Particle composition

Compositions of particles from sea-ice samples are depicted in Fig. 3a. For all samples, the dominant compositions were inorganic matter, accounting for 65% of all particles on average. Although MPs were minor components, they were detected from all ice subsections except for the middle part of Core 4. Composition ratios of MPs were 0–17%, with an average of 5%.

**Fig. 5:**
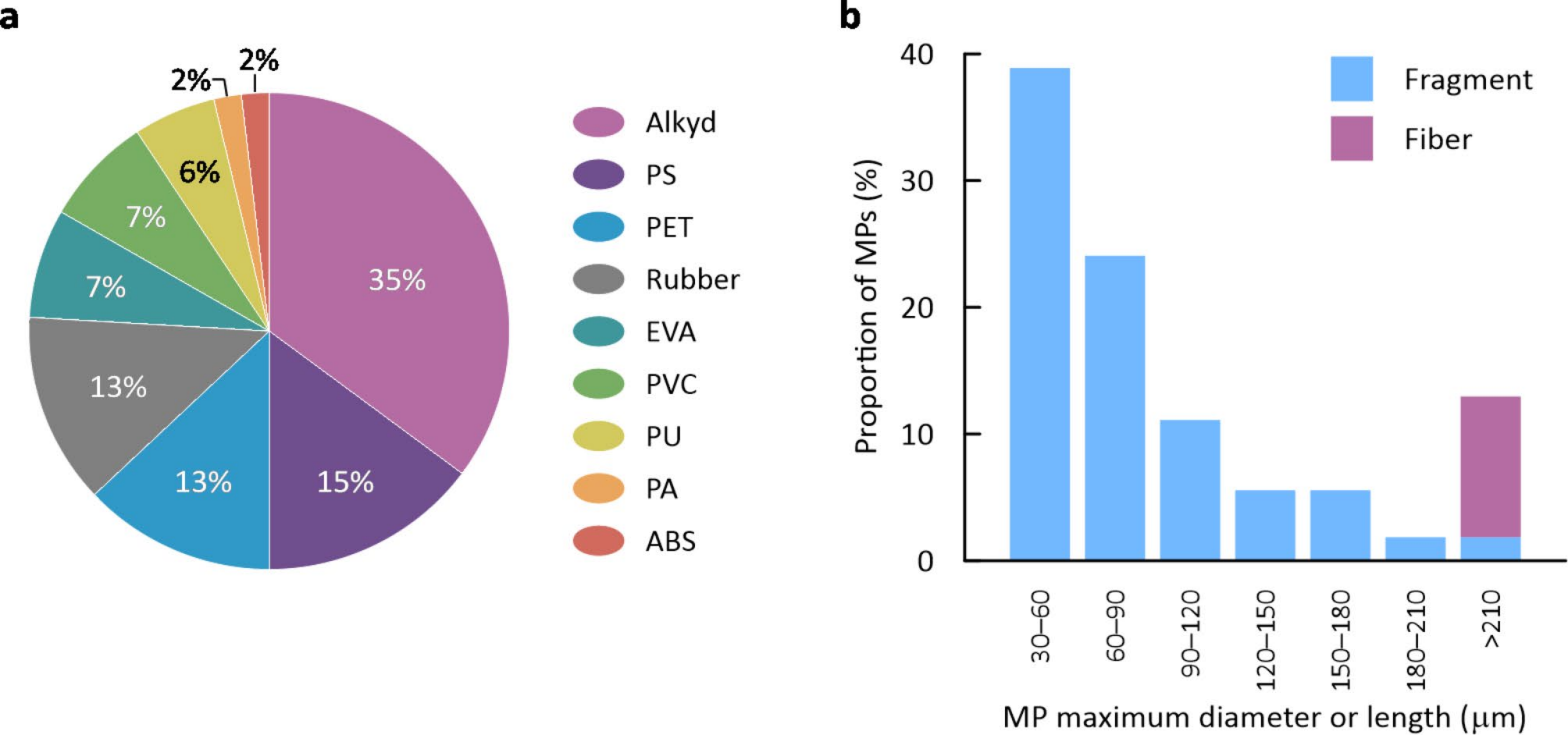
Data of microplastics from all sea-ice cores (*n* = 55). (**a**) Polymer compositions of MPs and (**b**) size distributions of fragment and fiber MPs.

Compositions of MPs in each ice subsection are depicted in Fig. 3b. Overall, plastic polymers of nine types were detected from microparticles: alkyd, polystyrene (PS), polyethylene terephthalate (PET), rubber, ethylene-vinyl acetate (EVA), polyvinyl chloride (PVC), polyurethane (PU), polyamide (PA), and acrylonitrile butadiene styrene (ABS). Although MP types depend strongly on ice subsections (Fig. 3b), this variation might be partly attributed to the small sample sizes (*n* in Fig. 3b). MP compositions are discussed later based on values averaged over all sea-ice cores (Fig. 5a).

**Fig. 6:**
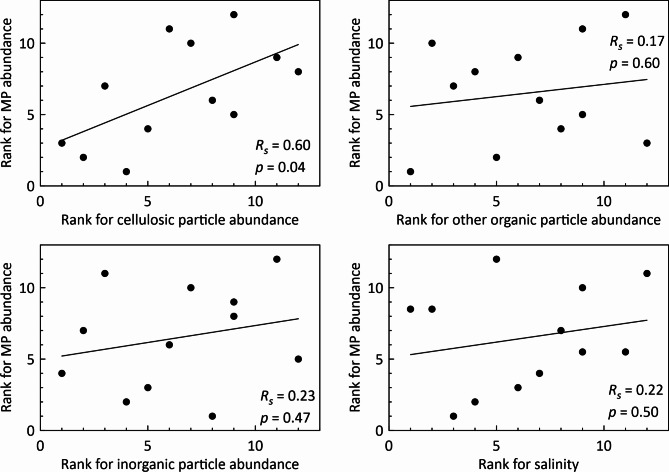
Scatter plots of ranks for MP abundance and other variables. *R*_*s*_ and *p* respectively denote Spearman’s rank correlation coefficient and *p*-value (significance). Solid lines in the figures are regression lines.

### Microplastic abundance

The highest concentration of MPs (60 particles/L) was observed for the lower part of Core 4, although no MP was found in the middle part of the same core (Table 1). The average value and the coefficient of variation of the MP abundances were, respectively, 21 particles/L and 79%.

### Microplastic composition and size

Observed frequencies of MPs from sea-ice samples are portrayed in Fig. 4 for various MP types in different size classes. The averaged values of polymer compositions of MPs are presented in Fig. 5a. Alkyd, PS, PET, and rubber were the dominant compositions of MPs in the sea-ice samples, accounting for 76%. Among these components, alkyd and rubber particles were observed in all ice cores, whereas PS and PET particles were detected only in specific cores (Figs. 3b and 4).

As a general trend, MPs were observed in the lower layers with high frequency (Fig. 4). In addition, MPs were mainly in the 30–120 μm size range (Fig. 4). Size distributions of fragment and fiber MPs are presented in Fig. 5b. The MPs from sea-ice samples were mostly fragment-shaped, accounting for 89%. More than 83% of these fragment MPs were of less than 120 μm maximum diameter, but all fiber MPs were longer than 210 μm. Fragment MPs consisted of all types of plastic polymers except for PA, whereas fiber MPs exclusively comprised PET and PA.

## Discussion

Overall, vertical profiles of MP abundances have indicated higher occurrence of MPs in the lower sections of ice: composition ratios of MPs were invariably the highest in lower parts of cores (Fig. 3a). Moreover, the average value of MP abundances in the lower layers (33 particles/L) was higher than those in the middle ones (13 particles/L) and upper ones (19 particles/L). These findings might be attributable to the growth region and drift path of sea ice^10^. As described above, the ices investigated in this study are regarded as advected from the northern part of the Sea of Okhotsk during its growth. Assuming that sea ice grows downward as a rule, the observed distributions of MPs imply that the degree of MP contamination in surface water of the sea is greater in the south. In fact, the Okhotsk coast of Hokkaido is more populated. For that reason, human activities such as fishery, tourism, and wastewater disposal are non-negligible local sources of MPs^11^. Another explanation is the redistribution of MPs in the ice matrix. It is generally believed that MPs are located selectively within the brines of sea ice^10,15,20^. Because of the gravity effect and downward motions of brine over time^37^, MPs can be relocated towards lower parts of the ice through brine channels.

The features of MPs in sea-ice reported to date differ considerably among the reported descriptions^9–15,20,21^. Although Zhang and others pointed out in their review article^38^ that this diversity might be attributable more to differences in sampling and analytical methods used than to actual regional differences, we strove to extract meaningful information by comparing our results with those reported from earlier studies^9–15^.

Obbard et al.^9^ reported that the abundance of MPs excluding rayon (cellulosic fiber) in Arctic Sea ice cores was, on average, approximately 45 particles/L. von Friesen et al.^11^ found approximately 60 MP particles per liter of melted sea ice from Svalbard. These values obtained by experimentation similarly to ours (microscopic observation + micro-FTIR analysis; targeted size range larger than several tens of micrometers) are of the same order of magnitude as the averaged MP abundance observed in our ice cores. The most frequent polymer type we detected was alkyd (Fig. 5a). The presence of alkyd particles as a major polymer component was confirmed also in Antarctic sea ice^14^. Alkyd resins are used widely in marine paints^39^. Other dominant polymer types examined for this work were PS, PET, and rubber (Fig. 5a). These compositions were observable in global sea-ice samples^10,13–15^.

The size distributions of fragment and fiber MPs observed for this study (Fig. 5b) are consistent with general trends reported from earlier works. The number of MPs observed generally increases concomitantly with decreasing size^10,14,15^. In terms of morphology, as observed from this work (Fig. 5b), fragments were the dominant shape for small microparticles (25–300 μm), whereas fiber shapes were common in larger size classes in MPs from first-year sea ice of the Novik Bay^15^. von Friesen et al.^11^ analyzed anthropogenic microparticles including MPs in both seawater and sea ice from Svalbard. They found that “fragment” particle morphology was dominant in sea ice, as in our case (Fig. 5b), whereas “fibers” were generally dominant in seawater. As Chubarenko^40^ pointed out, fiber MPs, since they are long and flexible, can be effectively captured within the brine channels network. Based on analyses of the depth profiles and size distributions of fragment and fiber MPs in the ice from Novik Bay, Chubarenko et al.^15^ have suggested preferential leakage of small (short) fiber MPs (< 300 μm) with brines during sea ice desalination, resulting in the observed dominance of fragmentary shapes in the small size range. This phenomenon might be a cause of the lack of small fiber MPs (< 210 μm) in our observations (Fig. 5b).

When considering the mechanism of MP incorporation into sea ice, suspension freezing of sediments (mostly mineral particles)^41,42^ has often been presented as an analogical phenomenon^9–15,20^. Sediment incorporation into sea ice is regarded as occurring mainly through underwater interaction between frazil ice and resuspended sediment^43^. However, our results demonstrate that the distribution of MPs and that of inorganic particles were independent (Fig. 6), implying considerable difference in the kinetics of entrainment into ice between MPs and sediments (mineral particles). No relation between MP concentrations and salinities was confirmed (Fig. 6), although the coexistence of MPs with brine is suspected, as described earlier. Significant positive correlation (*p*-value less than 0.05) was found only between MP and cellulosic-particle abundances (Fig. 6), possibly because of their similarities in origins (human activities): as described above, our cellulosic particles include human-modified fibers (e.g. Fig. 2j and t), but it is difficult in many cases to distinguish human-modified cellulose from natural cellulose based only on their FTIR spectra and features^36^. Some studies^10,13,14,40^ have suggested biological processes as factors controlling MP enrichment in sea ice: sticky extracellular polymeric substances (EPS) produced by ice algae can mediate MP incorporation into sea ice^44^, also, biofilm formation on MPs can modify particle buoyancy and therefore influence the fate of their suspension freezing^45^, although this hypothesis cannot be verified based on the dataset used for this study.

The complicated natures of the interaction of MPs with water solutions and forming ice have been shown by results of recent laboratory works. Pradel et al.^46^ simulated the sea ice growth by progressively freezing a saline solution containing MPs and nanoplastics (NPs). They found size-dependent behavior of particle incorporation into ice: MPs are retained in ice, whereas NPs are expulsed from it. Considerable amounts of plastic particles, such as PET, with density greater than that of seawater have been found in every study of natural sea ice^9–15,21^, including ours. Chubarenko et al.^47^ conducted freezing experiments of fresh and salty water containing heavy plastic particles (HPP). They demonstrated that the formation of (or aggregation with) gas bubbles at the particles in water can drive HPP to float in a water column, leading to HPP capture in ice. Chen et al.^48^ examined the entrainment and enrichment of MPs in ice formation processes under varying turbulence conditions. They found that high rotation speed in freshwater enhanced the entrainment of hydrophobic MPs, whereas high turbulence in saline water inhibited the incorporation of all types of MPs into ice, probably because of the loose structure of ice in a saline solution, which allows the exchange of MPs between ice and water. Very recently, the same research group^49^ investigated interactions between aquatic dissolved organic matter (DOM) and MPs and also the roles of DOM in the entrainment behaviors of MPs under freezing conditions. Their experimentally obtained results showed that the presence of humic acid sodium (HA) significantly altered the zeta potential (the charge which develops at the interface between a solid surface and its liquid medium) and wettability (contact angle for a water droplet on a material surface) of MPs. In most cases, the entrainment of MPs in ice was improved after their interaction with HA, probably because of surface damage caused by physical abrasion and ultraviolet radiation.

To elucidate the mechanism of suspension freezing of MPs, and to assess the probable relocation of MPs in the ice matrix after entrainment, systematic field work for multi-parametric measurements of sea ice in different growing (shrinking) stages and further experimental studies are both necessary.

## Data Availability

The datasets used or analyzed for this study are available from the corresponding author on reasonable request.
